# Assessment of sleep quality and its relationship with stress and anxiety, and attitudes toward sleep among adults in Riyadh, Saudi Arabia

**DOI:** 10.1097/MD.0000000000047708

**Published:** 2026-02-13

**Authors:** Rayan A. Qutob, Rahaf Almutairi, Hassan Alshamrani, Abdulrahman Aldawood, Kadi Alsubaie, Ghayda Aldosari, Abdullah Alaryni, Abdullah Alghamdi, Khalid I. AlHussaini, Abdulrahman Alanazi, Yousef Alammari, Mosaad Almegren, Fahad Ali Faqihi, Maaz Mohamed Salih, Khaled Aied Alharbi

**Affiliations:** a Department of Internal Medicine, College of Medicine, Imam Mohammad Ibn Saud Islamic University (IMSIU), Riyadh, Saudi Arabia; b College of Medicine, Imam Mohammad Ibn Saud Islamic University (IMSIU), Riyadh, Saudi Arabia; c Department of Critical Care Medicine, Dr. Sulaiman Al Habib Medical Group, Riyadh, Saudi Arabia.

**Keywords:** anxiety, quality, sleep, stress

## Abstract

Sleep is an essential element in every human being’s life, as it impacts the quality of an individual’s life. When a person’s sleep is uncomfortable and irregular, this leads to the possibility of exposure to many mental and physical illnesses that affect activity. This study aimed to measure the quality of sleep and its relationship to stress and anxiety, and what practices and attitudes that people do regarding their sleep. This is a cross-sectional study was employed to assess sleep quality and its relationship with stress and anxiety, and attitudes toward sleep among adults in Riyadh, Saudi Arabia between December 2024 and April 2025. Data were collected via a self-administered survey consisting of 5 validated questionnaires (satisfaction with life scale, hospital anxiety and depression scale, perceived stress scale [PSS], dysfunctional beliefs and attitudes about sleep [DBAS], and Pittsburgh sleep quality index [PSQI]). Logistic regression was used to find the influencing factors for each outcome. The study involved 687 participants. Married individuals were less likely to experience anxiety compared to singles (OR = 0.21, 95% CI: 0.1–0.45, *P* < .001). Older age was significantly associated with higher odds of elevated DBAS and poor PSQI scores. Individuals aged 61 and above had increased odds of dysfunctional beliefs about sleep (OR = 6.00, 95% CI: 1.25–28.74, *P* = .025) and poor sleep quality (OR = 22.65, 95% CI: 4.37–117.39, *P* < .001) compared to those age 18 to 20. Individuals with higher levels of DBAS, PSS, and anxiety (hospital anxiety and depression scale) were significantly associated with poorer sleep quality, as measured by PSQI score. Specifically, higher level of DBAS increased the odds of poor sleep (OR = 1.01, 95% CI:1.00–1.02, *P* = .02). Similar with stress, 1 point increase in the PSS scale, increased the poor quality of sleeping (OR = 1.16, 95% CI: 1.08–1.24, *P* < .001), while the anxiety level increased the odds of poor quality of sleeping 14% (OR = 1.14, 95% CI: 1.08–1.19, *P* = .001). This study emphasizes a robust relationship between inadequate sleep quality and psychological factors. A variety of demographic factors, including age, gender, marital status, nationality, and employment, are identified as substantial contributors.

## 1. Introduction

Sleep is an essential element in every human being’s life, as it impacts the quality of an individual’s life. When a person’s sleep is uncomfortable and irregular, this leads to the possibility of exposure to many mental and physical illnesses that affect activity.^[1-6]^ Poor sleep is a global problem due to its association with many diseases and complications, such as its impact on mental abilities, mood, weakness, fatigue, psychological health, increased incidence of heart and respiratory diseases, their impact on the immune system, and their association with chronic diseases such as diabetes and other diseases.^[7]^ When individual is exposed to some changes, whether in mood or behavior, this leads him to feel some negative feelings, which makes him stressful.^[8,9]^ While anxiety is a feeling of danger and tension towards various situations where the person is in a constant state of alert and worried about different things.^[10]^ There’s a clear link between sleep problems, stress, and anxiety, as each can be a cause of the other. For example, stress can sometimes lead to sleeplessness or insomnia. On the other hand, poor sleep can also cause psychological health problems, as excessive sleep can lead to anxiety and worrying. Both problems are also linked to elevated levels of the cortisol hormone, which puts the body in a state of stress and instability.^[11-13]^ Anxiety and stress are one of the main reasons for reducing the quality of sleep, as they reduce relaxation and increase the duration of wakefulness during sleep. When a change occurs in sleep patterns, this leads to a deterioration in human health, putting the person at risk for contracting multiple chronic diseases. Therefore, the opposite is true, as when stress and anxiety reduce then this leads to improving the quality of sleep and thus affecting the quality of life of the person.^[14-18]^ Attitudes towards sleep vary, as they include a person’s ideas about the importance of sleep and the number of hours needed to sleep. They also include an assessment of the quality of sleep and the person’s allocation of a specific time for sleep within his daily schedule.^[19]^ There are many bad habits that some people follow and lead to irregular sleep patterns and reduce the effectiveness of sleep, including not having a regular sleep schedule, sleeping for a long time during naps, drinking many stimulants such as coffee and others, smoking, drinking alcohol, eating before get to bed, using the phone before sleep, and not sleeping in a suitable environment that stimulates the melatonin hormone, which helps with sleep.^[20]^ In Riyadh, Saudi Arabia, people have many problems related to sleep which include using electronic devices for long time,^[21]^ lack of awareness of healthy sleeping habits,^[22]^ eating unhealthy foods,^[23]^ and taking a nap during the day,^[24,25]^ all of these factors were associated with decreasing the quality of sleeping. In general, there is a lack of research related to sleep problems and the factors that affect them in Riyadh, Saudi Arabia. Therefore, this study aimed to measure the quality of sleep and its relationship to stress and anxiety, and what practices and attitudes that people do regarding their sleep.

## 2. Materials and methods

### 2.1. Study design

This is a cross-sectional study was employed to assess sleep quality and its relationship with stress and anxiety, and attitudes toward sleep among adults in Riyadh, Saudi Arabia between December 2024 and April 2025.

### 2.2. Study population

The study population included adults aged above 18 in the general public in Riyadh city, Saudi Arabia. This research did not exclude participants based on their gender or other socioeconomic characteristics.

### 2.3. Participants recruitment

This research utilized a convenience sample technique to enroll participants. This research employed an online survey. For digital questionnaire. The questionnaire link was disseminated via social media sites such as Facebook and WhatsApp. The invitation letter for the questionnaire emphasized the study’s objective and inclusion criteria. Only participants who fulfilled the inclusion criteria were solicited to engage in the study.

### 2.4. Questionnaires

Data were collected via a self-administered survey consisting of 5 validated questionnaires (satisfaction with life scale [SWLS], hospital anxiety and depression scale [HADS], perceived stress scale [PSS], dysfunctional beliefs and attitudes about sleep [DBAS], and Pittsburgh sleep quality index [PSQI]).^[26-30]^ SWLS is comprised of 5-items that measure global life satisfaction – a cognitive component of subjective well-being, with each item scored on a 7-point Likert scale (1 = strongly disagree to 7 = strongly agree), resulting in total scores between 5 and 35; elevated scores signify increased life satisfaction. The HADS consists of 14 items including anxiety and depression subscales, with each item scored from 0 to 3. This yields subscale scores from 0 to 21; scores of 8 to 10 indicate borderline cases, while scores of 11 or above suggest probable clinical anxiety or depression. The perceived stress scale, comprised of 10-items assess perceived stress over the preceding month, with questions evaluated on a 5-point scale (0 = never to 4 = very often), resulting in total scores ranging from 0 to 40; elevated scores indicate more perceived stress. The dysfunctional views and attitudes about sleep comprises of 16 items, assesses maladaptive sleep-related cognitions, with responses evaluated on a 0 to 10 scale, where elevated scores indicate greater dysfunctional views regarding sleep. The PSQI is a 19-item instrument evaluating sleep quality over the preceding month across 7 dimensions (e.g., sleep latency, duration, disturbances), each rated from 0 to 3; the component scores are aggregated to produce a global score ranging from 0 to 21, with scores exceeding 5 signifying inadequate sleep quality. Besides, the study instrument included demographics questions such as age, gender, nationality, marital status, education level, occupation, and living area.

### 2.5. Ethical approval

Ethical approval for this research was obtained from the Institutional Review Board (IRB) in Al-Imam Muhammad Ibn Saud Islamic University (project number 754/2024).

### 2.6. Sample size

Using a confidence interval of 95%, a standard deviation of 0.5, and a margin of error of 5%, the required sample size was 385 participants.

### 2.7. Data analysis

The data were analyzed with SPSS version 29 (IBM Corp., Armonk). Demographic characteristics of the study population such as the gender, and age group were presented as frequency and percentage. The continuous data such as the scores were presented as the mean and standard deviation. To assess the normality of the data, the Kolmogrov–Smirnov test was performed, and since the data met the parametric assumptions, independent *t*-test and the analysis of variance tests were performed when applicable. For multiple group comparisons, Tukey post hoc test was applied, and the results were evaluated accordingly. The scores were categorized based on the literature cut off point for each scale. Logistic regression was used to find the influencing factor for each outcome separately. Odds ratios (ORs) and 95% confidence intervals (CI) were shown. A *P* value < .05 considered as significant level for all analysis.

## 3. Results

The study involved 687 participants. Most of them were aged 21 to 30 years (257, 37.4%), followed by 31 to 40 years (135, 19.7%), 18 to 20 years (89, 13.0%). Males accounted for 377 (54.9%) participants, while females were 310 (45.1%). The vast majority were Saudi nationals (662, 96.4%) compared to non-Saudis (25, 3.6%). Most participants were married (398, 57.9%), followed by single (253, 368%). Regarding education level, 471 (68.8%) held a university degree, 129 (18.8%) had a high school education, 78 (11.4%) had postgraduate degrees. In terms of occupation, 210 (30.6%) worked in the private sector, 193 (28.1%) were students, 180 (26.2%) were government employees. The majority lived in the middle regions (257, 37.4%), followed by the north (124, 18.0%), Table 1.

**Table 1:** Sociodemographic characteristics of study participants.

	N	%
Age		
18–20	89	13.0%
21–30	257	37.4%
31–40	135	19.7%
41–50	108	15.7%
51–60	71	10.3%
61-	27	3.9%
Gender		
Female	310	45.1%
Male	377	54.9%
Nationality		
Non-Saudi	25	3.6%
Saudi	662	96.4%
Marital status		
Single	253	36.8%
Married	398	57.9%
Divorced	23	3.3%
Widowed	13	1.9%
Education level		
Primary or less	3	0.4%
Middle	6	0.9%
High school	129	18.8%
University	471	68.6%
Post grad	78	11.4%
Occupation		
Non	63	9.2%
Student	193	28.1%
Government	180	26.2%
Private	210	30.6%
Retired	41	6.0%
Living area		
West	114	16.6%
North	124	18.0%
Middle	257	37.4%
South	113	16.4%
East	79	11.5%

### 3.1. Psychological and sleep measures profile

The total mean of the SWLT score was 22.7 out of 30 as maximum, achieved by 49.3%, while the HADS mean was 9.3 out of 39, achieved by 64.8%. The PSS, DBAS, and PSQI mean was 18.6, 63.8 and 13.2 out of 40, 155, and 42, respectively and the percentage was 56.9%, 54.5%, and 56.2%. The prevalence of stress and anxiety among patients with poor sleep quality were 67% and 84.7%, respectively. Participants aged 41 to 50 years had higher dysfunctional beliefs about sleep (DBAS: 70.88 ± 31.42) and poorer sleep quality (19.77 ± 12.43) compared to the younger participants aged 18 to 20 years (DBAS: 54.80 ± 33.53, PSQI: 8.56 ± 8.79) (*P* < .001). Widowed individuals reported the highest anxiety levels (HADS: 15.38 ± 5.72) and the highest DBAS scores (98.08 ± 34.99) among all marital groups (*P* < .001). Regarding education, those with only primary education or less showed elevated anxiety (HADS: 16.00 ± 8.00) and stress (PSS: 21.33 ± 0.58), along with highly dysfunctional beliefs (DBAS: 101.67 ± 14.19) (*P* < .001). In contrast, post graduate participants had the highest satisfaction with life (SWLS: 26.64 ± 3.39) and relatively better sleep quality (PSQI: 12.44 ± 8.52). Unemployed participants had higher anxiety (HADS: 13.76 ± 7.32), dysfunctional beliefs (DBAS: 75.70 ± 35.38), and poorer sleep quality (PSQI: 15.33 ± 9.44) than other occupational groups (*P* < .001), Table 2.

**Table 2:** Comparison of psychological and sleep measures across sociodemographic groups.

	SWLS total	*P* value	HADS total	*P*-value	PSS total	*P*-value	DBAS total	*P*-value	PSQI total	*P*-value
Age										
18–20	20.85 ± 5.87	.000***	7.55 ± 7.20	.000***	18.67 ± 4.69	.77	54.80 ± 33.53	.000***	8.56 ± 8.79	.001**
21–30	22.30 ± 5.83		9.29 ± 7.20		18.75 ± 3.38		60.11 ± 29.69		9.55 ± 8.07	
31–40	22.56 ± 5.97		8.39 ± 6.62		18.42 ± 3.30		59.37 ± 30.68		12.99 ± 9.50	
41–50	24.31 ± 5.44		9.86 ± 7.00		18.31 ± 3.23		70.88 ± 31.42		19.77 ± 12.43	
51–60	23.63 ± 5.15		11.68 ± 7.56		18.76 ± 3.06		74.73 ± 38.33		20.37 ± 11.74	
61-	24.22 ± 4.41		11.44 ± 6.36		19.22 ± 3.42		94.04 ± 33.61		20.07 ± 9.15	
Gender										
Female	22.50 ± 5.95	.42	9.13 ± 7.36	.55	18.50 ± 3.67	.38	63.27 ± 34.03	.69	12.67 ± 10.81	.21
Male	22.85 ± 5.61		9.46 ± 6.95		18.73 ± 3.36		64.26 ± 31.90		13.70 ± 10.64	
Nationality										
Non-Saudi	22.64 ± 4.86	.96	10.20 ± 5.55	.52	20.76 ± 3.27	.00***	73.84 ± 26.70	.12	17.16 ± 8.70	.06*
Saudi	22.70 ± 5.80		9.27 ± 7.19		18.55 ± 3.49		63.44 ± 33.02		13.09 ± 10.77	
Marital status										
Single	22.05 ± 5.37	.14	10.02 ± 7.85	.000***	19.00 ± 4.08	.04*	62.64 ± 32.06	.000***	10.39 ± 8.82	.001**
Married	23.07 ± 6.03		8.48 ± 6.57		18.34 ± 3.09		62.26 ± 32.95		14.94 ± 11.62	
Divorced	23.48 ± 5.37		12.26 ± 5.94		19.70 ± 3.50		84.13 ± 21.10		12.65 ± 8.80	
Widowed	22.46 ± 4.70		15.38 ± 5.72		18.08 ± 2.29		98.08 ± 34.99		17.46 ± 6.64	
Education level										
Primary or less	22.00 ± 6.93	.000***	16.00 ± 8.00	.000***	21.33 ± 0.58	.000***	101.67 ± 14.19	.000***	25.00 ± 5.00	.34
Middle	21.33 ± 6.28		11.33 ± 10.46		20.33 ± 3.08		75.67 ± 37.99		15.50 ± 13.03	
High school	20.68 ± 5.87		11.58 ± 8.82		19.57 ± 4.22		66.74 ± 34.50		12.94 ± 9.62	
University	22.62 ± 5.73		8.65 ± 6.67		18.38 ± 3.39		60.75 ± 32.96		13.35 ± 11.30	
Post grad	26.64 ± 3.39		9.10 ± 5.55		18.29 ± 2.58		75.13 ± 25.35		12.44 ± 8.52	
Occupation										
Non	21.11 ± 5.86	.000***	13.76 ± 7.32	.000***	18.83 ± 3.37	.06	75.70 ± 35.38	.000***	15.33 ± 9.44	.001**
Student	21.54 ± 5.66		9.03 ± 8.04		18.88 ± 4.21		58.28 ± 30.95		9.97 ± 9.11	
Government	23.30 ± 5.96		9.03 ± 6.51		18.97 ± 3.29		63.21 ± 33.56		13.45 ± 11.51	
Private	23.29 ± 5.66		8.17 ± 6.42		18.03 ± 2.96		61.60 ± 30.76		14.33 ± 11.29	
Retired	24.88 ± 4.15		10.85 ± 5.83		18.66 ± 3.31		85.59 ± 33.31		18.83 ± 8.83	
Living area										
West	22.42 ± 5.88	.10	8.54 ± 6.99	.000***	17.61 ± 2.86	.000***	61.66 ± 34.55	.000***	11.87 ± 11.07	.004**
North	23.35 ± 5.70		7.49 ± 6.01		17.75 ± 2.86		57.45 ± 26.20		12.80 ± 10.79	
Middle	22.71 ± 5.40		11.56 ± 7.49		20.11 ± 4.03		74.73 ± 33.05		15.11 ± 9.59	
South	23.22 ± 5.96		7.51 ± 6.33		17.45 ± 2.81		56.67 ± 32.85		10.88 ± 11.08	
East	21.27 ± 6.37		8.51 ± 7.10		18.32 ± 2.53		51.62 ± 29.34		13.16 ± 12.20	

DBAS = dysfunctional beliefs and attitudes about sleep, HADS = hospital anxiety and depression scale, PSQI = Pittsburgh sleep quality index, PSS = perceived stress scale, SWLS = satisfaction with life scale.

* *P* < .05.

** *P* < .01.

*** *P* < .001.

### 3.2. Predictors of satisfaction with life and anxiety

The regression analysis revealed that HADS score significantly increased with age, particularly among participants aged 21 to 30 (OR: 4.92, 95% CI: 2.22–10.91), 31 to 40 years (OR = 10.90, 95% CI: 3.81–31.21), and up to 51 to 60 years (OR: 31.21, 95% CI: 9.96–97.82, *P* < .001) compared to younger participants aged 18 to 20 years. Therefore, participants aged 21 to 30, 31 to 40, and 51 to 60 years were 4.9-fold, 10.9-fold, and 31.2-fold more likely to have higher level of anxiety compared to others. Males also had significantly higher anxiety than females (OR = 1.62, 95% CI: 1.09–2.24, *P* = .018) and were 60.0% more likely to have higher level of anxiety compared to females. Married individuals were 79.0% less likely to experience anxiety compared to singles (OR = 0.21, 95% CI: 0.1–0.45, *P* < .001). Employment status also played a role, with students, private sector employees, and retired individuals showing significantly lower anxiety levels compared to the unemployed group (*P* < .01), Table 3.

**Table 3:** Multivariate logistic regression analysis of predictors for satisfaction with life and anxiety.

	SWLS score		HADS score	
	OR (95% CI)	*P* value	OR (95% CI)	*P* value
Age				
18–20	Reference		Reference	
21–30	1.05 (0.54–2.05)	.891	4.92 (2.22–10.91)	<.001
31–40	1.10 (0.47–2.59)	.829	10.90 (3.81–31.21)	<.001
41–50	1.95 (0.81–4.70)	.137	17.97 (6.12–52.76)	<.001
51–60	1.22 (0.47–3.18)	.689	31.21 (9.96–97.82)	<.001
61-	0.47 (0.10–2.26)	.343	5.74 (1.09–30.34)	.040
Gender				
Female	Reference		Reference	
Male	0.85 (0.61–1.20)	.356	1.62 (1.09–2.42)	.018
Nationality				
Non-Saudi	Reference		Reference	
Saudi	1.79 (0.72–4.48)	.213	1.10 (0.44–2.76)	.843
Marital status				
Single	Reference		Reference	
Married	0.78 (0.43–1.42)	.414	0.21 (0.10–0.45)	<.001
Divorced	0.99 (0.35–2.78)	.985	0.47 (0.15–1.47)	.194
Widowed	0.25 (0.05–1.17)	.078	4.20 (0.72–24.45)	.110
Education level				
Primary or less	Reference		Reference	
Middle	0.24 (0.01–5.54)	.371	0.59 (0.02–21.92)	.777
High school	0.15 (0.01–2.25)	.169	1.26 (0.08–21.01)	.873
University	0.24 (0.02–3.48)	.293	0.38 (0.02–6.17)	.493
Post grad	0.90 (0.06–13.78)	.938	0.58 (0.03–9.87)	.703
Occupation				
Unemployed	Reference		Reference	
Student	1.06 (0.50–2.23)	.885	0.18 (0.08–0.42)	<.001
Government	1.31 (0.68–2.54)	.424	0.31 (0.15–0.66)	.002
Private	1.08 (0.56–2.08)	.811	0.21 (0.10–0.44)	<.001
Retired	4.30 (1.19–15.53)	.026	0.14 (0.04–0.50)	.002
Living area				
West	Reference		Reference	
North	1.37 (0.79–2.37)	.260	1.02 (0.52–1.97)	.963
Middle	0.99 (0.60–1.61)	.953	3.18 (1.80–5.60)	<.001
South	1.62 (0.93–2.82)	.091	0.81 (0.40–1.64)	.555
East	0.81 (0.43–1.51)	.507	1.09 (0.53–2.28)	.809

CI = confidence interaval, HADS = hospital anxiety and depression scale, OR = odds ratio, SWLS = satisfaction with life scale.

### 3.3. Predictors for perceived stress, dysfunctional beliefs about sleep, and sleep quality

Older age was significantly associated with higher odds of elevated DBAS and poor PSQI scores. Individuals aged 61 and above had 6-fold higher odds of dysfunctional beliefs about sleep (OR = 6.00, 95% CI: 1.25–28.74, *P* = .025) and 22.7-fold higher odds of having poor sleep quality (OR = 22.65, 95% CI: 4.37–117.39, *P* < .001) compared to those age 18 to 20 years. Being Saudi was significantly associated with 75.0% lower odds of having perceived stress (OR = 0.25, 95% CI: 0.10–0.66, *P* = .005) and 66.0% lower odds of having fewer dysfunctional beliefs (OR = 0.34, 95% CI:0.12–0.93, *P* = .035) compared to non-Saudis. Married individuals were 67.0% less likely to experience stress (OR = 0.33, 95% CI: 0.17–0.63, *P* = .001), 68.0% less likely to have dysfunctional beliefs (OR = 0.32, 95% CI: 0.17–60, *P* = .014) compared to singles. Individuals with higher levels of DBAS, PSS, and anxiety (HADS) were significantly associated with poorer sleep quality, as measured by PSQI score. Specifically, higher level of DBAS increased the odds of poor sleep (OR = 1.01, 95% CI:1.00–1.02, *P* = .02). Similar with stress, 1 point increase in the PSS scale, increased the poor quality of sleeping by 16.0% (OR = 1.16, 95% CI: 1.08–1.24, *P* < .001), while the anxiety level increased the odds of poor quality of sleeping by 14% (OR = 1.14, 95% CI: 1.08–1.19, *P* = .001), Table 4.

**Table 4:** Multivariate logistic regression analysis of predictors for perceived stress, dysfunctional beliefs about sleep, and sleep quality.

	PSS score		DBAS score		PSQI score	
	OR (95% CI)	*P* value	OR (95% CI)	*P* value	OR (95% CI)	*P* value
Age						
18–20	Reference		Reference		Reference	
21–30	1.25 (0.69–2.26)	.469	1.77 (0.97–3.21)	.061	0.95 (0.46–1.98)	.893
31–40	1.49 (0.64–3.45)	.354	2.97 (1.30–6.82)	.010	4.10 (1.49–11.27)	.006
41–50	1.59 (0.67–3.81)	.293	5.94 (2.52–14.02)	<.001	8.51 (2.94–24.66)	<.001
51–60	2.51 (0.99–6.37)	.053	3.59 (1.42–9.05)	.007	14.28 (4.33–47.07)	<.001
61-	4.70 (1.06–20.82)	.041	6.00 (1.25–28.74)	.025	12.50 (1.92–81.34)	.008
Gender						
Female	Reference		Reference		Reference	
Male	1.31 (0.92–1.86)	.135	0.91 (0.65–1.29)	.610	1.03 (0.69–1.54)	.867
Nationality						
Non-Saudi	Reference		Reference		Reference	
Saudi	0.25 (0.10–0.66)	.005	0.34 (0.12–0.93)	.035	0.67 (0.22–2.06)	.489
Marital status						
Single	Reference		Reference		Reference	
Married	0.33 (0.17–0.63)	.001	0.32 (0.17–0.60)	<.001	0.94 (0.43–2.06)	.886
Divorced	1.02 (0.34–3.09)	.973	2.61 (0.65–10.41)	.175	0.21 (0.06–0.74)	.016
Widowed	0.56 (0.13–2.37)	.434	1.18 (0.21–6.72)	.852	0.55 (0.10–3.10)	.502
Occupation						
Unemployed	Reference		Reference		Reference	
Student	0.35 (0.16–0.76)	.008	0.33 (0.16–0.71)	.005	1.87 (0.75–4.66)	.177
Government	1.06 (0.56–2.01)	.856	0.59 (0.31–1.12)	.104	0.65 (0.29–1.46)	.294
Private	0.30 (0.16–0.58)	<.001	0.44 (0.23–0.84)	.012	1.33 (0.61–2.90)	.477
Retired	0.18 (0.05–0.60)	.005	0.90 (0.27–3.04)	.868	0.61 (0.14–2.71)	.519
Living area						
West	Reference		Reference		Reference	
North	1.06 (0.59–1.92)	.844	1.28 (0.74–2.23)	.376	1.60 (0.84–3.05)	.155
Middle	4.14 (2.46–6.97)	<.001	2.63 (1.59–4.34)	<.001	1.46 (0.80–2.68)	.216
South	1.18 (0.65–2.15)	.589	0.97 (0.55–1.72)	.924	0.97 (0.49–1.92)	.936
East	1.76 (0.93–3.32)	.082	0.63 (0.33–1.20)	.162	1.52 (0.73–3.14)	.263
DBAS total					1.01 (1.00–1.02)	.024
PSS total					1.16 (1.08–1.24)	<.001
HADS total					1.14 (1.08–1.19)	<.001

DBAS = dysfunctional beliefs and attitudes about sleep, HADS = hospital anxiety and depression scale, PSQI = Pittsburgh sleep quality index, PSS = perceived stress scale.

## 4. Discussion

Sleep problems are considered widespread problems in recent times, so this study came to measure the quality of sleep and its relationship to some psychological conditions such as anxiety and stress and to know what people’s behaviors and beliefs are regarding sleep. This study examined this topic in Riyadh in the Kingdom of Saudi Arabia.

In this study, the prevalence of stress and anxiety among patients with poor sleep quality were 67% and 84.7%, respectively. In a study of Turkish citizens to measure the relationship between anxiety, stress, and sleep quality, it was noted that the majority of people with sleep disturbances suffered from moderate levels of anxiety and stress, which affected their sleep patterns.^[31]^ In another study that agreed with our findings, it was demonstrated that not getting enough sleep affects people’s mental abilities, as worrying and psychological distress were more prevalent among people who had sleep impairment.^[32]^

Moreover, HADS score significantly increased with age, particularly among participants aged 21 to 30 (OR: 4.92, 95% CI: 2.22–10.91), 31 to 40 years (OR = 10.90, 95% CI: 3.81–31.21), and up to 51 to 60 years (OR: 31.21, 95% CI: 9.96–97.82, *P* < .001 for all). Males also had significantly higher anxiety than females (OR = 1.62, 95% CI: 1.09–2.24, *P* = .018). In a French sample and using the same scale the results showed the increasing in age was associated with scale increases, which is consistent with our result in the case study. However, in contrast to our result from the same study women scored higher than men on the same scale.^[33]^ This was consistent with other studies, all of which highlighted those women scored higher than men.^[34]^

In this study, married individuals were found to be less likely to experience anxiety compared to singles (OR = 0.21, 95% CI: 0.1–0.45, *P* < .001). In a study conducted by Kader Maideen et al to determine the role of social status on anxiety, it became clear that the category in which anxiety was highest was the divorced, then the separated, then the widows, then the singles, that is, those who live alone or are not in a relationship, and the lowest cases were recorded among the married, which was consistent with our findings in this study.^[35]^ Another study on Saudi Arabia revealed that persons who are single more suffer from psychological tension and worrying about their future life compared with married.^[36]^ In our study, employment status also played a role, with students, private sector employees, and retired individuals showing significantly lower anxiety levels compared to the unemployed group (*P* < .01). This was aligned with a study conducted on Korean population to examine the relationship between employment status and mental well-being. It was found that people who were unemployed or without a job had more mental health difficulties than those who were employed.^[37-39]^

In this study, older age was significantly associated with higher odds of elevated DBAS and poor PSQI scores. Individuals aged 61 and above had increased odds of dysfunctional beliefs about sleep (OR = 6.00, 95% CI: 1.25–28.74, *P* = .025) and poor sleep quality (OR = 22.65, 95% CI: 4.37–117.39, *P* < .001) compared to those age 18 to 20. To measure people’s thoughts and opinions toward sleep, a scale DBAS was used, and to measure sleep efficiency, PSQI was used. In a study on the elderly, it became clear that negative feelings such as anxiety and stress are linked to strange beliefs about sleep among this group, unlike young people whose ideas are different. Therefore, in both groups, the quality of sleep is bad and good, respectively.^[40]^

Furthermore, being Saudi was significantly associated with lower perceived stress (OR = 0.25, 95% CI: 0.10–0.66, *P* = .005) and fewer dysfunctional beliefs (OR = 0.34, 95% CI:0.12–0.93, *P* = .035) compared to non-Saudis. Contrary to the results of our study, a study on Saudi students showed that they suffer from stress and poor sleep quality. Poor sleep quality was found in more than half of the sample, and its presence was linked to the presence of stress and anxiety.^[32]^ Married individuals were less likely to experience stress (OR = 0.33, 95% CI: 0.17–0.63, *P* = .001), dysfunctional beliefs (OR = 0.32, 95% CI: 0.17–60, *P* = .014). This is in contrast to a study conducted on a number of married people, which showed that their beliefs about sleep developed in an abnormal way compared to those who were separated or unmarried.^[41]^

In this study, individuals with higher levels of DBAS, PSS, and anxiety (HADS) were significantly associated with poorer sleep quality, as measured by PSQI score. Specifically, higher level of DBAS increased the odds of poor sleep (OR = 1.01, 95% CI:1.00–1.02, *P* = .02). Similar with stress, 1 point increase in the PSS scale, increased the poor quality of sleeping (OR = 1.16, 95% CI: 1.08–1.24, *P* < .001), while the anxiety level increased the odds of poor quality of sleeping 14% (OR = 1.14, 95% CI: 1.08–1.19, *P* = .001). Stress and anxiety are linked to sleep quality, as its presence affects beliefs and attitudes about sleep, which negatively affects sleep quality.^[42]^ Another study in Turkey also supported these findings.^[43,44]^

This study has limitations. The use of an online cross-sectional survey study design has limited generatability. Besides, self-administered survey study design is prone to reporting bias. The online cross-sectional survey study design using convenience sampling technique restricted the generatability of the study findings, the ability to examine causality among the study variables, and introduce selection bias. However, we believe that the large sample size in this research increase the generalizability of our findings. Moreover, this study is prone to reporting bias and social desirability as it utilized self-administered questionnaire. Besides, due to the use of online survey study design, we were not able to estimate the number of participants who were invited to participate; therefore, we cannot estimate the number of respondents or the response rate for this study, which increase the possibility of nonresponse bias. Therefore, the study findings should be interpreted carefully.

Based on the study findings, policymakers in the healthcare sector in Saudi Arabia are advised to prioritize effective sleep-health strategies for the general public. Primary healthcare centers should offer regular assessment and screening for sleep quality and mental health, specifically for high-risk population identified in this research (example: individuals aged 61 and above). Public health campaigns should enhance public awareness and knowledge on the importance of good-quality sleep. Moreover, work-related regulations should aim to regulate working hours by reducing sleep disruption among all workers through offering flexible shift settings.

## 5. Conclusion

This study emphasizes a robust relationship between inadequate sleep quality and psychological factors, including stress, anxiety, and dysfunctional beliefs about sleep. A variety of demographic factors, including age, gender, marital status, nationality, and employment, are identified as substantial contributors. These results emphasize the multifaceted character of sleep disturbances and the significance of addressing psychological well-being and social context in the management of sleep health. In order to evaluate the efficacy of targeted behavioral or psychosocial interventions and establish causal relationships, future research should investigate longitudinal and interventional designs. Furthermore, research that integrates qualitative insights and broader cultural or environmental factors may enhance our comprehension of the ways in which diverse populations confront and resolve sleep-related obstacles.

## Author contributions

**Conceptualization:** Rayan A. Qutob.

**Data curation:** Rayan A. Qutob.

**Formal analysis:** Rayan A. Qutob.

**Funding acquisition:** Rayan A. Qutob.

**Investigation:** Rayan A. Qutob, Rahaf Almutairi, Hassan Alshamrani, Abdulrahman Aldawood, Kadi Alsubaie, Ghayda Aldosari, Abdullah Alaryni, Abdullah Alghamdi, Khalid I. AlHussaini, Abdulrahman Alanazi, Yousef Alammari, Mosaad Almegren, Fahad Ali Faqihi, Maaz Mohamed Salih, Khaled Aied Alharbi.

**Methodology:** Rayan A. Qutob.

**Project administration:** Rayan A. Qutob.

**Resources:** Rayan A. Qutob, Rahaf Almutairi, Hassan Alshamrani, Abdulrahman Aldawood, Kadi Alsubaie, Ghayda Aldosari, Abdullah Alaryni, Abdullah Alghamdi, Khalid I. AlHussaini, Abdulrahman Alanazi, Yousef Alammari, Mosaad Almegren, Fahad Ali Faqihi, Maaz Mohamed Salih, Khaled Aied Alharbi.

**Software:** Rayan A. Qutob.

**Supervision:** Rayan A. Qutob.

**Validation:** Rayan A. Qutob.

**Visualization:** Rayan A. Qutob.

**Writing – original draft:** Rayan A. Qutob.

**Writing – review & editing:** Rayan A. Qutob, Rahaf Almutairi, Hassan Alshamrani, Abdulrahman Aldawood, Kadi Alsubaie, Ghayda Aldosari, Abdullah Alaryni, Abdullah Alghamdi, Khalid I. AlHussaini, Abdulrahman Alanazi, Yousef Alammari, Mosaad Almegren, Fahad Ali Faqihi, Maaz Mohamed Salih, Khaled Aied Alharbi.
